# Potentially More Out of Reach: Public Reporting Exacerbates Inequities in Home Health Access

**DOI:** 10.1111/1468-0009.12616

**Published:** 2023-03-24

**Authors:** SHEKINAH A. FASHAW‐WALTERS, MOMOTAZUR RAHMAN, GILBERT GEE, VINCENT MOR, MARICRUZ RIVERA‐HERNANDEZ, CERON FORD, KALI S. THOMAS

**Affiliations:** ^1^ School of Public Health University of Minnesota; ^2^ Center for Gerontology and Healthcare Research School of Public Health Brown University; ^3^ School of Public Health Brown University; ^4^ Fielding School of Public Health University of California at Los Angeles; ^5^ US Department of Veterans Affairs Medical Center Center of Innovation in Long‐Term Services and Supports

**Keywords:** home health, quality, racial inequities, public reporting, access

## Abstract

**Policy Points**

- Public reporting is associated with both mitigating and exacerbating inequities in high‐quality home health agency use for marginalized groups.
- Ensuring equitable access to home health requires taking a closer look at potentially inequitable policies to ensure that these policies are not inadvertently exacerbating disparities as home health public reporting potentially does.
- Targeted federal, state, and local interventions should focus on raising awareness about the five‐star quality ratings among marginalized populations for whom inequities have been exacerbated.

**Context:**

Literature suggests that public reporting of quality may have the unintended consequence of exacerbating disparities in access to high‐quality, long‐term care for older adults. The objective of this study is to evaluate the impact of the home health five‐star ratings on changes in high‐quality home health agency use by race, ethnicity, income status, and place‐based factors.

**Methods:**

We use data from the Outcome and Assessment Information Set, Medicare Enrollment Files, Care Compare, and American Community Survey to estimate differential access to high‐quality home health agencies between July 2014 and June 2017. To estimate the impact of the home health five‐star rating introduction on the use of high‐quality home health agencies, we use a longitudinal observational pretest–posttest design.

**Findings:**

After the introduction of the home health five‐star ratings in 2016, we found that adjusted rates of high‐quality home health agency use increased for all home health patients, except for Hispanic/Latine and Asian American/Pacific Islander patients. Additionally, we found that the disparity in high‐quality home health agency use between low‐income and higher‐income home health patients was exacerbated after the introduction of the five‐star quality ratings. We also observed that patients within predominantly Hispanic/Latine neighborhoods had a significant decrease in their use of high‐quality home health agencies, whereas patients in predominantly White and integrated neighborhoods had a significant increase in high‐quality home health agency use. Other neighborhoods experience a nonsignificant change in high‐quality home health agency use.

**Conclusions:**

Policymakers should be aware of the potential unintended consequences for implementing home health public reporting, specifically for Hispanic/Latine, Asian American/Pacific Islander, and low‐income home health patients, as well as patients residing in predominantly Hispanic/Latine neighborhoods. Targeted interventions should focus on raising awareness around the five‐star ratings.

Public reporting of quality information is expanding across the US health care system through the Centers for Medicare and Medicaid Services (CMS) “Care Compare” website (https://www.medicare.gov/care‐compare/). Care Compare is designed to improve provider accountability, quality transparency, and patient‐centered care, and its effects are well supported by the literature.^1^, ^2^, ^3^, ^4^ There are two proposed interconnected mechanisms for how public reporting works to improve quality: (1) provider response and (2) consumer response. Provider response refers to providers using the information to change their practices, improve their quality, and bolster their market share and reputation.^5^, ^6^, ^7^ Consumer response assumes that consumers will use the information to choose high‐quality providers, which in turn can influence the providers’ responses.^5^, ^6^

Despite the documented benefits of publicly reporting quality information, it has been suggested that public reporting may have unintended consequences for patients, professionals, and institutions as a whole and contributes to increased disparities.^4^ These unintended consequences include possible selection bias (e.g., cream‐skimming, cherry‐picking, and sorting), in which providers will either intentionally or unintentionally exclude patients based on race, ethnicity, or income status in order to improve their quality rating.^4^, ^8^, ^9^ These unintended consequences are believed to exacerbate disparities.^9^ Prior research suggests that following a new requirement to report mortality rates for cardiac procedures, hospitals avoided high‐risk patients and providers discriminated against patients on the basis of illness severity and race.^8^, ^10^ These discriminatory actions ultimately led to reduced care for patients in need, greater rates of adverse outcomes, and increased costs to the Medicare program.^8^, ^10^ Prior research has also documented high‐quality nursing homes selectively admitted more profitable residents while avoiding Medicaid residents and that socioeconomic disparities in the use of high‐quality nursing homes were exacerbated by the introduction of the nursing home five‐star rating system.^9^, ^11^, ^12^ Despite research focused on nursing homes and hospitals warning against the negative unintended consequences of market‐based reforms, less is known about the impact of public reporting of home health quality on access.^13^

Home health is an important source of care for older adults. Medicare home health patients on average are older, poorer, and sicker than other Medicare beneficiaries.^14^ Over 25% of Medicare home health beneficiaries are older than 85 years of age, two‐thirds are living below 200% of the federal poverty line (FPL), and over half have five or more chronic conditions.^14^, ^15^ In 2018, there were about 3.4 million homebound Medicare fee‐for‐service home health patients receiving care from about 11,500 home health agencies and who accounted for $18 billion dollars in spending for the Medicare program.^16^ Between 2002 and 2018, the number of home health fee‐for‐service patients has increased by about 37%. As the demand for home health continues to grow, it is increasingly important to ensure access to high‐quality care for all patients.^16^

Because home health is a health care service that is uniquely delivered in patients’ homes rather than in a centralized, physical location like a nursing home, concerns about the unintended consequences of public reporting may be even greater as providers and consumers respond to quality information. For example, changes in provider service areas/supply (e.g., provider market entries and exits) and shifts in consumer choices (e.g., switching provider) are more feasible (e.g., lower opportunity/financial costs) than would be observed in other settings.^17^, ^18^ In fact, prior research suggests the original introduction of the Home Health Compare website in 2003 (predecessor to the Care Compare website) slightly increased home health agency market exits in poorer neighborhoods^17^ and slightly increased the use of high‐quality home health agencies for consumers.^18^ However, these two studies examined the introduction of the original Home Health Compare ratings close to two decades ago that reported many different individual measures of quality rather than the more simplified, composite five‐star measure of quality introduced in 2015. To date, there have been very few studies on the impact of the home health five‐star quality ratings and none to our knowledge on the five‐star quality ratings’ impact on disparities in home health access.

Furthermore, even though previous research findings show that disparities in access to high‐quality home health exists within and across neighborhoods,^19^ to date, no study has examined the impact of the five‐star quality ratings introduction on this within‐ and between‐neighborhood phenomenon. It might be assumed that living in the same neighborhood would afford residents equal access to high‐quality home health providers, but the evidence has shown otherwise, and we aim to better understand the role of public reporting in this access disparity.^19^ In addition, looking across or between neighborhoods is equally important because of how historic structures of inequity have systematically limited access to high‐quality hospitals, primary care physicians, nursing homes, and community‐based long‐term services and supports (e.g., assisted living).^20^, ^21^, ^22^, ^23^, ^24^, ^25^, ^26^ Understanding the within‐ and between‐neighborhood mechanisms for disparities in access to high‐quality home health agencies and their relationship with the introduction of home health five‐star quality ratings will help guide policy and intervention development aimed to advance health equity.

Given recent research findings showing that there are racial, ethnic, and socioeconomic individual‐level disparities in receiving care from high‐quality home health agencies and that these disparities are strongly correlated with place‐based (neighborhood) factors,^19^ the objective of this study is to evaluate the impact of the introduction of Care Compare five‐star ratings for home health on changes in the likelihood of receiving care from a high‐quality home health agency by race, ethnicity, income status, and place. We do this by asking two questions: (1) Does the introduction of the five‐star ratings change access to high‐quality home health agencies within neighborhoods for Black, Hispanic/Latine, Asian American/Pacific Islander, American Indian/Alaska Native, and low‐income home health patients? and (2) Does the introduction of the five‐star ratings change access to high‐quality home health agencies for beneficiaries living in neighborhoods with more Black, Hispanic/Latine, Asian American/Pacific Islander, American Indian/Alaska Native, or socioeconomically disadvantaged residents? Drawing on prior literature, we test two hypotheses. First, we hypothesize that following the introduction of the home health five‐star ratings, Black, Hispanic/Latine, Asian American/Pacific Islander, American Indian/Alaska Native, and low‐income home health patients will have a smaller increase in their use of high‐quality home health agencies as compared with their White and higher‐income counterparts within the same neighborhoods. Last, we hypothesize that following the introduction of the home health five‐star ratings, neighborhoods that have more minoritized residents and those with more socioeconomic disadvantage will have a smaller increase in their access to high‐quality home health agencies than predominantly White neighborhoods and more socioeconomically advantaged areas.

## Methods

### Data Sources

The data came from the 2014–2017 Medicare Beneficiary Summary File, the 2014–2017 Enrollment Database, the 2014–2017 Outcome and Assessment Information Set (OASIS), and the 2016–2018 Care Compare website. Geographical data came from the 2015 American Community Survey 5‐year estimates and the 2013 National Center for Health Statistics Urban–Rural Classification Scheme for Counties.

The Medicare Beneficiary Summary File contains demographic characteristics, enrollment information, Zone Improvement Plan (ZIP) codes, and the social security administration standard county code. The Enrollment Database is the CMS database of record for Medicare beneficiary enrollment information; it contains ZIP codes that correspond with each month of the year. Medicare‐certified home health agencies are required to submit OASIS assessments for all Medicare beneficiaries receiving skilled home health services. We used the OASIS to identify individual home health patients, the home health agency serving them, and other individual‐level information (e.g., living arrangements). These data are linked to the Medicare Beneficiary Summary File using the beneficiary identification (ID) number.

Since July 2015, all Medicare‐certified home health agencies have a publicly reported star rating, which is updated quarterly on the CMS Care Compare website (https://www.medicare.gov/care‐compare). The home health agencies’ star ratings are linked to beneficiary data using the Medicare provider number for each home health agency. The quality‐of‐care five‐star quality ratings, which range from one to five with half‐star intervals, are used to characterize home health agency quality, our primary outcome variable. The 2016–2018 five‐star quality ratings are calculated as a composite measure by the CMS using various process and outcome measures from the OASIS and Medicare claims. The 2016–2018 five‐star quality ratings include nine measures of quality: (1) timely initiation of care, (2) drug education on all medications provided to patient/caregiver, (3) influenza immunization received for current flu season, (4) improvement in ambulation, (5) improvement in bed transferring, (6) improvement in bathing, (7) improvement in pain interfering with activity, (8) improvement in shortness of breath, and (9) acute care hospitalizations. Details on how the five‐star quality ratings are calculated are publicly available on the CMS's website.^27^

Last, neighborhood racial composition and poverty status (derived from the poverty rate) is collated from the American Community Survey 5‐year estimates available through data.census.gov, and county rurality is derived from the National Center for Health Statistics Urban–Rural Classification Scheme for Counties.^28^ The neighborhood details are described further below. These data are linked to the beneficiary‐level data using ZIP Code Tabulation Areas and Social Security Administration standard county codes.

### Study Design and Sample

This study estimates the impact of the introduction of the home health five‐star quality ratings on the use of high‐quality home health agencies using a longitudinal observational pretest–posttest design.

Our sample consists of Black, Hispanic/Latine, Asian American/Pacific Islander, American Indian/Alaskan Native, and White Medicare‐enrolled, community‐dwelling home health patients, ages 65 years and older, with a start‐of‐care assessment during our study period. The sample is limited to home health patients from the 48 contiguous states. We exclude home health patients residing in congregate housing (e.g., assisted living; *n* = 945,600 beneficiaries) because beneficiaries in congregate settings may have limited control over which home health agency they use. We also exclude OASIS patient assessments without a matching provider number in the Care Compare data (*n* = 28,008 beneficiaries and 28,386 assessments); characteristics of the excluded observations can be seen in the Appendix 1. Our final analytic sample consists of 6,136,961 beneficiaries with 7,001,512 unique start‐of‐care assessments during the 3‐year study period.

### Outcome

Our outcome variable is receiving care from a high‐quality home health agency. This variable is dichotomous and identifies home health agencies as high quality if their average five‐star quality rating is greater than 3.5 stars across 12 quarters of data (January 2016 to December 2018); otherwise, home health agencies are identified as non–high‐quality home health agencies. Home health agencies with an average of 3.5 stars were chosen as high‐quality home health agencies because the CMS recognize above average quality as having greater than three stars.^29^ One consistent star rating was applied in the pre‐ and poststar rating periods so as not to inadvertently measure changes in the star rating over time. Across 12 quarters of data, it is unlikely that quality changes substantially; however, the star ratings themselves may change, and we did not want our results to be driven by such changes. Consequently, we chose to interpret the average rating as a proxy for the steady state of overall quality. As a sensitivity analysis, we vary how we calculate the quality outcome (see sensitivity analyses below) and explore how the average high‐quality status changes over time (Appendix 2).

### Explanatory Variables

To identify non‐Hispanic Black, Hispanic/Latine, Asian American/Pacific Islander, American Indian/Alaska Native, and non‐Hispanic White home health patients, we use the self‐reported race variable from the OASIS.^30^ All racial/ethnic groups are mutually exclusive. Similar to previous studies, a patient's low‐income status is determined by dual enrollment in Medicare and Medicaid and/or participation in Medicare Part‐D low‐income, cost‐sharing subsidy (LIS).^19^ We use the Part‐D LIS to identify low‐income patients because it has a more generous eligibility threshold than Medicaid eligibility, does not vary by state, and was therefore more sensitive to capturing low‐income Medicare beneficiaries.

Neighborhoods are defined by the ZIP Code Tabulation Areas, which are mapped onto the beneficiaries’ home ZIP code using the Uniform Data Set mapper (https://udsmapper.org/). We use the beneficiary ZIP code (from the Enrollment Database) that corresponds with the month of their OASIS start‐of‐care assessment. We include two neighborhood characteristics: neighborhood racial composition and poverty status.

Neighborhood racial composition is operationalized using a categorical variable of racial predominance for each neighborhood and consists of seven mutually exclusive categories: (1) predominantly White, (2) predominantly Black, (3) predominantly Hispanic/Latine, (4) predominantly Asian American/Pacific Islander, (5) predominantly American Indian/Alaska Native, (6) predominantly minority, and (7) integrated. Neighborhoods that are predominantly White, Black, Hispanic/Latine, Asian American/Pacific Islander, or American Indian/Alaska Native are defined as ≥65% White, Black, Hispanic/Latine, Asian American/Pacific Islander, or American Indian/Alaska residents, respectively, and according to the American Community Survey. Minority neighborhoods are operationalized as ≥65% minoritized residents (but not predominantly any other racial/ethnic minority group), whereas integrated neighborhoods are ZIP Code Tabulation Areas that do not fit in the first six categories. These cut points were chosen to align with work by Usher and colleagues that used place‐based measures of race and poverty.^31^ Neighborhood poverty status is operationalized using a quintile of the percentage of residents who live below 200% of the FPL. Neighborhoods are labeled as being in the lowest, second, middle, fourth, or highest poverty quintile.

### Covariates

A number of covariates are also included in the study: sex, age, Medicare Advantage enrollment, living alone, region of the country, and county rurality. We control for the sex and age of home health patients with data from the Medicare Beneficiary Summary File. We also include beneficiaries’ Medicare Advantage enrollment status at the time of the home health episode as defined in the Medicare Beneficiary Summary File to control for utilization differences that exist between Traditional Medicare and Medicare Advantage enrollees.^32^, ^33^ We measure living alone as a reflection of home health patients’ social support using the patient living situation variable in the OASIS.^34^ Last, region of the country is developed using the Medicare Beneficiary Summary File data on state, and county rurality is derived from the National Center for Health Statistics Urban–Rural Classification Scheme.^28^ Controlling for region and rurality is important because of geographic variability in access to home health and distribution or racial and ethnic groups.

### Analysis

We use data from July 2014 to June 2017 (with a two‐quarter lag for when the five‐star ratings were partially implemented) and have ten quarters of data. The prestar period included quarter 3 (July) 2014 through quarter 2 (June) 2015, whereas the poststar period included quarter 1 (January) 2016 through quarter 2 2017. All analyses are conducted at the user‐quarter level. We only include the first start‐of‐care assessment in each quarter per beneficiary. Summary statistics are calculated for patient and neighborhood characteristics in the prestar and poststar periods.

To address our objectives, we conduct two separate analyses. The objective of our first analysis is to assess whether the likelihood of high‐quality home health agency use differs for minoritized and low‐income patients residing in the same neighborhood and how these differences changed in the postperiod. Prior literature shows that one of the key drivers of racial differences in access to high‐quality care includes neighborhood characteristics.^19^ Thus, we estimate the relationship between the introduction of the home health agency five‐star ratings and changes in high‐quality home health agency use at the patient level using linear probability models with ZIP Code Tabulation Area (neighborhood) fixed effects and adjustments for sex, age, Medicare Advantage status, and living alone. We use the Stata 17 margins command to then predict the adjusted rate of high‐quality home health agency use before and after the five‐star ratings using the equations below:^35^

(1) $$\begin{array}{ccc} Y_{izt} & = & \beta_{0}\;+\;\beta_{1}Post_{t}+\beta_{2}Race_{i}+\beta_{3}Income_{i}+\beta_{4}\left(Post_{t}\times \;Race_{i}\right)\;\\ & & +\;\beta_{5}Patient_{i}+\delta_{z}+\;\upsilon_{izt} \end{array}$$

(2) $$\begin{array}{ccc} \;Y_{izt} & = & \beta_{0}\;+\;\beta_{1}Post_{t}+\beta_{2}Income_{i}+\beta_{3}Race_{i}+\beta_{4}\left(Post_{t}\times \;Income_{i}\right)\;\\ & & +\;\beta_{5}Patient_{i}+\delta_{z}+\;\upsilon_{izt}, \end{array}$$

Where $Y_{izt}$ is a binary outcome indicating use of a high‐quality home health agency for individual *i* residing in neighborhood *z* at time *t*. $Post_{t}$ indicates the time period after the introduction of five‐star public reporting. $Race_{i}$ is a vector of four dummy variables indicating different races, where White patients serve as the benchmark racial group because of their higher rate of high‐quality home health agency use. $Income_{i}$ is a binary indicator for persons with low income. Our main variables of interest are the interactions of introduction of five‐star public reporting (*Post*) and a patient's *Race* and *Income* status. β_4_ is a parameter (a vector of four parameters in equation 1 and a scalar in equation 2) of interest and represents the change in likelihood of using a high‐quality home health agency for different racial groups and income categories in the postperiod. $Patient_{i}$ is a vector of patient characteristics listed in the covariates section. $\delta_{z}$ is a vector of ZIP Code Tabulation Area (neighborhood) fixed effects. $\upsilon_{izt}$ is the error term. Using ZIP code fixed effects allows us to look at variation in access to high‐quality home health agencies within the same neighborhood, where it might be assumed that access is equal.^19^

Second, to examine the relationship between the introduction of the home health agency five‐star ratings and changes in high‐quality home health agency use by neighborhood racial composition and poverty, we use linear probability models that adjust for sex, age, Medicare Advantage status, living alone, region of the country, and rurality using the following model:

(3) $$\begin{array}{ccc} Y_{it} & = & \beta_{0}\;+\;\beta_{1}Post_{t}+\beta_{2}Place_{i}+\beta_{3}\left(Post_{t}\times \;Place_{i}\right)\;\\ & & +\;\beta_{4}Patient_{i}+\;\upsilon_{it}. \end{array}$$

We then plot the changes in high‐quality home health agency use (β_3_) by the neighborhood characteristics (i.e., racial composition and poverty). These models represent the impact of the five‐star rating on access to high‐quality home health agencies between differing neighborhood types, which is important to examine given prior literature and the principal role of place in access to high‐quality home health agencies for minoritized patients.^19^

### Sensitivity Analyses

We conducted several sensitivity analyses to ensure the robustness of our results. First, we conducted sensitivity analyses with three alternatively specified high‐quality outcome variables: (1) the average of the star ratings in 2016 is high quality, (2) the median of the star ratings across 12 quarters is high quality, and (3) the mode of the star ratings across 12 quarters is high quality. Second, given that Home Health Value‐Based Purchasing (HHVBP) was introduced in 2016 (our postperiod), providing a direct financial incentive to home health agencies for improving their quality and including measures found in the five‐star quality rating, we conducted a sensitivity analysis to account for the introduction of HHVBP. We include an indicator variable for the nine HHVBP pilot states as a covariate within the regression analysis. Last, given the relatively large size of our neighborhoods (identified by ZIP Code Tabulation Areas), we conduct a sensitivity analysis lowering the neighborhood racial composition measurement from >65% to >50%.

## Results

Of the 6,136,961 home health Medicare beneficiaries with start‐of‐care assessments during our study period, there are a total of 7,001,512 assessments across the ten quarters of data. A total of 2,840,634 assessments are in the four‐quarter prestar period and 4,160,878 assessments in the six‐quarter poststar period (Table 1). The population of home health patients is similar before and after the introduction of the five‐star ratings. The percentage of home health patients receiving care from high‐quality home health agencies rose by less than 1 percentage point (pp) in the postperiod, increasing from an average of 47.2% to 47.7%. Racial distributions remained relatively similar in the prestar and poststar periods. The percentage of low‐income patients decreased slightly from 26.6% in the prestar period to 26.0% in the poststar period. All other individual‐level covariates remained relatively similar over time. On average, home health patients in the preperiod lived in neighborhoods where 34.5% of the residents lived below 200% of the FPL; in the postperiod, this decreased to 34.3%. Most home health patients resided in predominantly White neighborhoods, followed by integrated, minority, Hispanic/Latine, Black, Asian American/Pacific Islander, and American Indian/Alaska Native neighborhoods—in that order.

**Table 1:** Characteristics of Home Health Patients and Patients’ Neighborhoods, Before and After the Introduction of the Home Health Five‐Star Ratings

	**Before Stars Ratings**	**After Stars Ratings**
Number of start‐of‐care assessments	2,840,634	4,160,878
Quality rating categories, %		
Unrated	0.3	0.2
Low quality	7.6	7.0
Average quality	45.0	45.1
High quality	47.2	47.7
Race/ethnicity, %		
White	78.6	78.5
Black	11.3	11.4
Hispanic/Latine	7.3	7.0
Asian American/Pacific Islander	2.5	2.6
American Indian/Alaska Native	0.4	0.4
Other demographics		
Low income, %	26.6	26.0
Average age, mean (SD)	78.96 (8.33)	78.79 (8.33)
Female, %	60.6	59.8
Medicare Advantage, %	27.0	28.6
Living alone, %	28.2	27.5
Neighborhood poverty		
Percent below 200% FPL, mean (SD)	34.5 (15.3)	34.3 (15.2)
Neighborhood racial composition, %		
≥65% White	59.8	59.9
≥65% Black	3.9	3.8
≥65% Hispanic/Latine	4.8	4.4
≥65% Asian American/Pacific Islander	0.1	0.1
≥65% American Indian/Alaska Native	0.04	0.04
≥65% minority	7.5	7.6
Integrated	24.9	24.2
Neighborhood region, %		
Northeast	21.1	20.2
Midwest	20.8	20.5
South	41.4	41.8
West	16.7	17.5
Neighborhood urban–rural classification, %		
Large central	28.6	28.5
Large fringe	24.7	24.8
Medium	21.8	21.8
Small	9.2	9.3
Micropolitan	9.0	8.9
Noncore	6.8	6.7

Abbreviations: SD, standard deviation; ZIP, Zone Improvement Plan.

The authors’ analysis is of data from the 2014–2017 Medicare Beneficiary Summary File (MBSF), the 2014–2017 Outcome and Assessment Information Set (OASIS), and the 2016–2018 Care Compare website. The geographical data came from the 2015 American Community Survey (ACS) 5‐year estimates and the 2013 National Center for Health Statistics (NCHS) Urban–Rural Classification Scheme for Counties. The data came from the start‐of‐care assessment and represent 2,692,656 unique patients before the star ratings and 2,825,796 unique patients after the star ratings. High‐quality home health agencies (HHAs) have >3.5 stars. Non–high‐quality HHAs include unrated, low‐star (1‐2.5 stars), and average‐star (3‐3.5) HHAs. Low income identifies a beneficiary as having dual enrollment in Medicare and Medicaid and/or participation in Medicare Part‐D low‐income, cost‐sharing subsidy. Medicare Advantage enrollment status as defined in the MBSF. Living Alone was measured using the OASIS variable for living situation. Neighborhood is defined by the ZIP Code Tabulation Area. Neighborhood poverty status is operationalized using a quintile of the percentage of residents who live below 200% of the federal poverty line (FPL).

**Figure 1:**
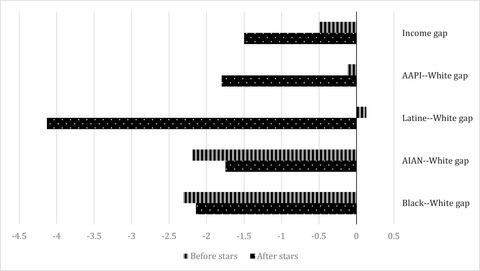
Within‐Neighborhood Individual‐level Racial, Ethnic, and Socioeconomic Gaps in High‐Quality Home Health Agency Use Before and After Public Reporting. Abbreviations: AAPI, Asian American/Pacific Islander; AIAN, American Indian/Alaska Native. The authors’ analysis is of data from the 2014–2017 Medicare Beneficiary Summary File (MBSF), the 2014–2017 Outcome and Assessment Information Set (OASIS), and the 2016–2018 Care Compare website. *N* = 7,001,512 for start‐of‐care assessments. The unit of analysis is at the person‐quarter level. The adjusted analysis is adjusted for sex, age, Medicare Advantage status, and living alone at the time of the assessment and includes neighborhood fixed effects (FEs). High‐quality home health agencies have >3.5 stars. Low‐income identifies a beneficiary as having dual enrollment in Medicare and Medicaid and/or participation in Medicare Part‐D low‐income, cost‐sharing subsidy. The Medicare Advantage enrollment status is as defined in the MBSF. Living alone was measured using the OASIS variable for living situation.

*Within Neighborhoods*. We present the actual and predicted rates of high‐quality home health agency use in the pre‐ and poststar periods by race, ethnicity, and income status in Table 2. Relative to the prestar period, there is a 1.1% relative increase in the use of high‐quality home health agencies in the poststar period. However, this increase is not experienced equally across all groups. White, Black, and American Indian/Alaska Native home health patients experienced a 1.8%, 2.6%, and 4.9% relative increase in the use of high‐quality home health agencies, respectively. Hispanic/Latine and Asian American/Pacific Islander patients experienced a 9.5% and 0.5% relative decrease in the use of high‐quality home health agencies, respectively. Higher income home health patients experienced a 1.7% relative increase in high‐quality home health agency use, whereas low‐income home health patients experienced a 0.5% decrease. When including neighborhood fixed effects and adjusting for sex, age, Medicare Advantage status, and living alone at the time of assessment, there continues to be an overall relative increase in the use of high‐quality home health agencies (1.3%; *p* = 0.000) that is experienced differentially across racial, ethnic, and socioeconomic groups. Hispanic/Latine and Asian American/Pacific Islander home health patients are the only groups of patients that experience a statistically significant relative decrease in high‐quality home health agency use (−7.0%; *p* = 0.000 and −1.6%; *p* = 0.000, respectively). White home health patients have a 2.0% (*p* = 0.000) relative increase, Black home health patients have a 2.5% (*p* = 0.000) relative increase in high‐quality home health agency use, and American Indian/Alaska Native home health patients experienced a 3.1% (*p* = 0.006) relative increase in high‐quality home health agency use. Higher income home health patients have a 1.9% (*p* = 0.000) increase in high‐quality home health agency use, and low‐income home health patients have a 0.3% (*p* = 0.074) relative decrease. These findings are also displayed visually in Figure 1B in Appendix 3 of the supplementary materials.

**Table 2:** Actual and Predicted Use of High‐Quality Home Health Agencies Before and After the Introduction of the Home Health Five‐Star Rating by Race, Ethnicity, and Income Status

**Unadjusted Percentage of High‐Quality Home Health Agency Use**				
**Demographic Characteristics**	**Before Stars**	**After Stars**	**Differences, pp**	**Percentage Change, %**
Race				
White	48.8	49.7	0.9	1.8
Black	41.7	42.8	1.1	2.6
Hispanic/Latine	39.8	36.0	−3.8	−9.5
Asian American/Pacific Islander	43.4	43.2	−0.2	−0.5
American Indian/Alaska Native	39.0	40.9	1.9	4.9
Income status				
Higher income	48.4	49.2	0.8	1.7
Low income	43.7	43.5	−0.2	−0.5
Overall	47.2	47.7	0.5	1.1

**Adjusted Predicted Percentage of High‐Quality Home Health Agency Use with FEs**				
**Demographic Characteristics**	**Before Stars, % (95% CI)**	**After Stars, % (95% CI)**	**Difference**	**Percentage Change**
Race				
White	47.38	48.33	0.95 pp	2.01%
	(47.32, 47.43)	(48.28, 48.37)	(0.88, 1.02)	(1.86, 2.15)
Black	45.07	46.19	1.12 pp	2.49%
	(44.90, 45.23)	(46.05, 46.33)	(0.93, 1.31)	(2.07, 2.90)
Hispanic/Latine	47.51	44.20	−3.31 pp	−6.97%
	(47.30, 47.72)	(44.02, 44.39)	(−3.55, −3.06)	(−7.51, −6.41)
Asian American/	47.26	46.52	−0.74 pp	−1.57%
Pacific Islander	(46.93, 47.59)	(46.25, 46.79)	(−1.15, −0.33)	(−2.45, −0.69)
American Indian/Alaska Native	45.19	46.58	1.39 pp	3.08%
	(44.41, 45.97)	(45.94, 47.21)	(0.40, 2.37)	(0.90, 5.16)
Income status				
Higher income	47.24	48.13	0.89 pp	1.88%
	(47.18, 47.30)	(48.08, 48.18)	(0.81, 0.96)	(1.72, 2.03)
Low income	46.75	46.63	−0.12 pp	−0.26%
	(46.65, 46.85)	(46.55, 46.72)	(−0.24, 0.01)	(−0.51, 0.02)
Overall	47.11	47.73	0.62 pp	1.32%
	(47.06, 47.16)	(47.70, 47.78)	(0.56, 0.69)	(1.19, 1.46)

Abbreviations: CI, confidence interval; FE, fixed effect; pp, percentage points.

The authors’ analysis is of data from the 2014–2017 Medicare Beneficiary Summary File (MBSF), the 2014–2017 Outcome and Assessment Information Set (OASIS), and the 2016–2018 Care Compare website. *N* = 7,001,512 for start‐of‐care assessments. The unit of analysis is at the person‐quarter level. The adjusted analysis is adjusted for sex, age, Medicare Advantage status, and living alone at the time of the assessment and includes neighborhood FEs. High‐quality home health agencies have >3.5 stars. Low income identifies a beneficiary as having dual enrollment in Medicare and Medicaid and/or participation in Medicare Part‐D low‐income, cost‐sharing subsidy. Medicare Advantage enrollment status is as defined in the MBSF. Living alone was measured using the OASIS variable for living situation.

In Figure 1, we present findings on the change in disparities between home health patients with more privilege (i.e., White or higher income) and those who are more marginalized (e.g., Black, Hispanic/Latine, low income). We find that the gap in high‐quality home health agency use between low‐income and higher‐income home health patients increases after public reporting, along with the gaps between White home health patients and Asian American/Pacific Islander and Hispanic/Latine home health patients. However, disparities in high‐quality home health agency use decreased among White home health patients and those who identify as American Indian/Alaska Native or Black.

**Figure 2:**
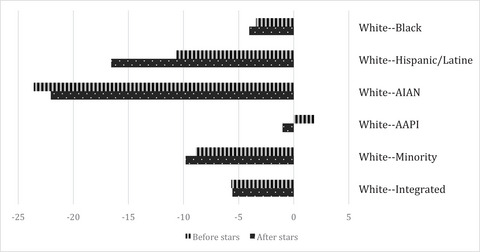
Between‐Neighborhood Gaps in High‐Quality Home Health Agency Use Before and After Public Reporting and by Neighborhood Racial Composition. Abbreviations: AAPI, Asian American/Pacific Islander; AIAN, American Indian/Alaska Native; ZIP, Zone Improvement Plan. The authors’ analysis is of data from the 2014–2017 Medicare Beneficiary Summary File (MBSF), the 2014–2017 Outcome and Assessment Information Set (OASIS), and the 2016–2018 Care Compare website. Geographical data came from the 2015 American Community Survey (ACS) 5‐year estimates and the 2013 National Center for Health Statistics (NCHS) Urban–Rural Classification Scheme for Counties. *N* = 7,001,512 for start‐of‐care assessments. High‐quality home health agencies (HHAs) have >3.5 stars. The unit of analysis is at the person‐quarter level. The adjusted analysis is adjusted for sex, age, Medicare Advantage status, living alone, region of the country, rurality, and neighborhood poverty. Neighborhood is defined by the ZIP Code Tabulation Area (ZCTA). Neighborhoods that are predominately White, Black, Hispanic/Latine, AAPI, or AIAN must be made up of ≥65% White, Black, Hispanic/Latine, AAPI, or AIAN residents, respectively, and according to the ACS. Minority neighborhoods must be made up of ≥65% minority residents (but not be predominately Black, Hispanic/Latine, AAPI, or AIAN), whereas integrated neighborhoods are ZCTAs that do not fit in the first six categories.

**Figure 3:**
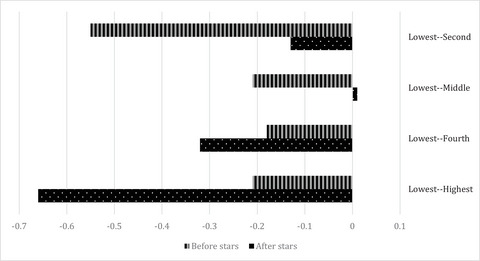
Between‐Neighborhood Gaps in High‐Quality Home Health Agency Use Before and After Public Reporting and by Neighborhood Poverty Status. Abbreviations: AAPI, Asian American/Pacific Islander; AIAN, American Indian/Alaska Native; ZIP, Zone Improvement Plan. The authors’ analysis is of data from the 2014–2017 Medicare Beneficiary Summary File (MBSF), the 2014–2017 Outcome and Assessment Information Set (OASIS), and the 2016–2018 Care Compare website. Geographical data came from the 2015 American Community Survey (ACS) 5‐year estimates and the 2013 National Center for Health Statistics (NCHS) Urban–Rural Classification Scheme for Counties. *N* = 7,001,512 for the start‐of‐care assessments. High‐quality home health agencies (HHAs) have >3.5 stars. The unit of analysis is at the person‐quarter level. The adjusted analysis is adjusted for sex, age, Medicare Advantage status, living alone, region of the country, rurality, and neighborhood racial composition. Neighborhood is defined by the ZIP Code Tabulation Area. Neighborhood poverty status is operationalized using a quintile of the percentage of residents who live below 200% of the federal poverty line (FPL).

*Across Neighborhoods*. Adjusting for sex, age, Medicare Advantage status, living alone, region of the country, and rurality at the time of assessment, we find an increase in the use of high‐quality home health agencies following the five‐star ratings that varies by neighborhood racial composition and poverty status (Figures 2 and 3 in Appendix 3). Home health agency patients in predominantly White and integrated neighborhoods experience statistically significant increases in use of high‐quality home health agencies in the poststar period (0.9 pp; *p* = 0.000 and 1.0 pp; *p* = 0.000, respectively). Conversely, home health patients in predominantly Hispanic/Latine neighborhoods experience a significant decrease (−5.1 pp; *p* = 0.000) in their use of high‐quality home health agencies. Home health patients in predominantly Black neighborhoods experience a 0.2‐pp (*p* = 0.202) increase, patients in American Indian/Alaska Native neighborhoods experience a 2.4‐pp (*p* = 0.222) increase, patients in predominantly Asian American/Pacific Islander neighborhoods experience a −2‐pp (*p* = 0.104) change, and patients in minority neighborhoods experience a −0.1‐pp (*p* = 0.621) change in their use of high‐quality home health agencies. Home health patients across neighborhoods, regardless of the poverty quintile, experience a statistically significant increase in high‐quality home health agency use, except for those in neighborhoods in the highest poverty quintile (0.05 pp; *p* = 0.533). Home health patients in neighborhoods in the lowest poverty quintile have an increase of 0.5 pp, those in the second poverty quintile have a change of 0.92 pp, those living in neighborhoods in the middle poverty quintile have an increase of 0.72 pp, and those in the fourth poverty quintile have a 0.36‐pp change in high‐quality home health agency use.

Figures 2 and 3 show the change in disparities among neighborhoods following the star rating. Compared with predominantly White neighborhoods, predominantly Black, Hispanic/Latine, Asian American/Pacific Islander, and minority neighborhoods saw an increase in the across‐neighborhood disparity in high‐quality home health agency use. When compared with neighborhoods in the lowest poverty quintile, disparities in high‐quality home health agency use decreased for neighborhoods in the second and middle poverty quartiles. Conversely, the disparity in high‐quality home health agency use increased for neighborhoods in the fourth and highest poverty quartile when compared with neighborhoods in the lowest poverty quartile.

*Sensitivity Analysis*. We completed several robustness checks and found that the overall patterns remained similar. Results can be found in the supplementary materials/Appendices 1–7 along with information about the stability of the star rating over time.

## Discussion

Our study finds that even though the home health five‐star quality ratings may be associated with an increase in high‐quality home health agency use overall, the five‐star ratings may have an inequitable impact across individual racial, ethnic, and socioeconomic groups, as well as neighborhoods. The findings from this study may be patient, provider, and/or structurally/institutionally driven; however, it is difficult to parse out and disaggregate the mechanisms underlying our findings. This discussion explores potential next steps for mitigating and further investigating the observed disparities found within this study.

It has been assumed that public reporting may mitigate disparities in quality;^36^, ^37^, ^38^ however, prior research suggests it can also exacerbate disparities.^4^, ^6^, ^13^ Public reporting of care quality has the potential to reduce disparities in access to high‐quality care by improving patients’ choice of provider and adding transparency and accountability to care processes.^38^ Conversely, public reporting may exacerbate or allow disparities to persist by failing to consider and correct for the existing patient, provider, and systemic structures of inequity.^13^ Our findings suggest that home health public reporting might simultaneously mitigate disparities for some groups while exacerbating disparities for others.

Following the introduction of the five‐star quality ratings, we find evidence of a reduction in high‐quality home health agency use disparities for Black and American Indian/Alaska Native home health patients when compared with their White counterparts. Our finding concerning Black home health patients is not surprising given recent findings that Black home health patients were more likely to select high‐quality home health agencies following public reporting, potentially because of the accessibility of the summary star rating^39^; this may also be the case for American Indian/Alaska Native home health patients. Prior to 2015/2016, the CMS publicly reported home health quality information without a summary star rating; creating the summary rating may have made the information more accessible for marginalized populations by bolstering its interpretation.^40^, ^41^ The slight narrowing of the gap in high‐quality home health agency use may also be related to large secular trends in home health (e.g., increases in the supply of home health agencies)^42^ that may have been accompanied by improvements in management, outreach, and competition. Future research should further evaluate why Black and American Indian/Alaska Native home health patients experience an increase in high‐quality home health agency use to better determine what interventions may translate to other settings and for other populations of color.

Despite a statistically insignificant change in the high‐quality home health agency for low‐income patients, the change by income status was significantly different, and the socioeconomic disparity in high‐quality home health agency use between higher‐income and low‐income home health patients is exacerbated following the introduction of the five‐star quality ratings. This observed disparity in high‐quality home health agency use could be caused by differences in the use of publicly reported information or home health agencies’ preferences to serve higher‐income patients.^6^, ^8^, ^9^, ^10^, ^12^, ^17^, ^43^, ^44^, ^45^, ^46^, ^47^, ^48^ Interventions targeting low‐income home health patients should focus on improving high‐quality home health agency access by providing more information on how to use the five‐star quality ratings and/or by incentivizing home health agencies to serve the low‐income population more readily.

Similar mechanisms to those that may have caused the socioeconomic disparity may also be responsible for the increased disparity among Hispanic/Latine, Asian American/Pacific Islander, and White home health patients. One patient‐driven mechanism to consider is that Hispanic/Latine and Asian American/Pacific Islander home health patients may not have been using the star ratings to help them choose a home health agency in the same way as other home health patients potentially used the five‐star ratings.^49^ Research posits that marginalized groups use and understand the star ratings less than more privileged groups.^9^, ^12^, ^45^, ^46^ Related to home health specifically, the literature surrounding the actual use of home health publicly reported quality is mixed.^39^, ^50^ Recent quantitative research by Schwartz and colleagues found that Black and lower‐income Medicare home health patients experienced the greatest increases in choosing high‐quality home health agencies following the introduction of the star ratings as compared with their White and high‐income counterparts.^39^ Conversely, earlier qualitative work by Baier and colleagues found that consumers and hospital case managers were unaware of the home health agency publicly reported quality and in turn did not use it in their decision making.^50^ Our findings support these mixed results, whereas it appears that some marginalized groups may potentially use the stars (e.g., Black patients) and others may not (e.g., low‐income or Hispanic/Latine patients), allowing for disparities to persist and worsen. Interventions targeting Hispanic/Latine and Asian American/Pacific Islander home health patients should focus on raising awareness around the five‐star ratings and clarifying other useful resources to aid in the selection of high‐quality services, as there may be a lack of familiarity with available resources.^51^

There may have also been shifts in the types of services used and needed by Hispanic/Latine and Asian American/Pacific Islander patients or the services provided by home health agencies. Future work should disaggregate home health use by the types of services utilized to evaluate if there were changes after the introduction of the five‐star ratings. It is also possible that potential language barriers may have been related to the decrease in use of high‐quality home health agencies for Hispanic/Latine and Asian American/Pacific Islander home health patients given that 4 million Medicare beneficiaries are limited in English proficiency.^52^

Future work needs to examine if high‐quality home health agencies have multilingual direct care workers and resources available to support racially and ethnically diverse communities that may have non‐English language preferences, especially given proposed quality metrics that would deem direct care workers “speaking with an accent” as providing lower quality care.^53^ Research in home health has documented higher workloads for home health nurses and therapists who provide care to patients with a non‐English language preference and the need for adequate reimbursement for interpreter services and higher workloads.^54^, ^55^ Recent research also found that language preference is associated with the type and intensity of home health care services for people living with dementia,^56^ whereas language discordance between patient and provider can lead to increased readmission rates.^57^ The CMS should consider reimbursing for interpreter services and making language preference information (e.g., whether an agency provides interpreters or multilingual providers) publicly available on the CMS Care Compare website, as this may be a priority in choosing care for racially and ethnically diverse populations.

Another potential reason for our findings may be that publicly reported composite star ratings may not report measures of interest to Hispanic/Latine and Asian American/Pacific Islander populations, so this study may not be measuring the quality that all groups care about.^58^, ^59^ Future work should approach this study with varied measures of quality, including the patient experience star ratings. In fact, recent research has found a weak correlation between patient experience star ratings and the quality‐of‐care star ratings used in this study.^60^ Understanding if the patient experience star ratings capture measures/domains that are more important to patients’ home health agency selection can be key to developing better measures of quality and improving care experiences and outcomes for all home health patients.

Furthermore, Hispanic/Latine and Asian American/Pacific Islander home health patients may be missing home health care opportunities from higher quality home health agencies at a higher rate after the introduction of the five‐star ratings than they did prior to the ratings. Research found that in 2016, only 54% of patients discharged to home health actually received home health within 14 days, and this rate was lowest among Hispanic/Latine patients; however, this study did not determine whether this lower receipt of care was related to the 2016 star ratings.^43^ Examining “missed” home health care opportunities and the impact of public reporting is an important future question. Li and colleagues posited that the observed disparity in timely home health use may have been a result of home health agencies being unable or unwilling to serve socioeconomically disadvantaged patients because of their potentially complex care needs and/or personal preferences.^43^, ^44^, ^45^, ^48^, ^61^ We think that these same issues identified by Li and colleagues may be impacting our current findings for Hispanic/Latine and Asian American/Pacific Islander home health patients.^43^ Smith and colleagues had similar findings that showed Asian American/Pacific Islander patients discharged from a diabetes‐related hospitalization to home health were also less likely to start care within the suggested 14 days following discharge.^62^ It is possible that home health agencies may have begun avoiding Hispanic/Latine and Asian American/Pacific Islander patients and communities because of perceptions about their risk behaviors and compliance with medical advice and their desire to protect and/or improve their quality ratings.^7^, ^43^, ^44^, ^45^, ^48^ Researchers have found selection bias on the part of providers based on health risks, race, and socioeconomic status.^4^, ^6^, ^8^, ^9^, ^10^, ^11^, ^47^, ^63^, ^64^ Future work should also consider the role of relationships between discharging hospitals and home health agencies because research suggests that existing provider relationships (both formal and informal) may also impact the selection of home health agencies.^50^ These existing relationships may lead to certain populations using or not using high‐quality home health agencies, possibly depending on the quality of the discharging hospital or their relationship with the receiving home health agency.

Based on our findings that most neighborhoods saw an increase in high‐quality home health agency use, except for predominantly Hispanic/Latine and Asian American/Pacific Islander, and that across‐neighborhood disparities—by race and socioeconomic status—were exacerbated following the star ratings, it may be the case that home health agencies are no longer serving or may be avoiding certain communities that may be considered “riskier” as compared with predominantly White neighborhoods. We see a similar pattern in the nursing home industry, in which fewer high‐quality nursing homes are in low‐income neighborhoods^65^ and more generally in health care in which we know that high‐quality providers are less likely to serve minoritized neighborhoods.^20^, ^21^, ^22^, ^23^, ^24^, ^25^, ^66^ This issue of not serving minoritized and lower‐income neighborhoods is not only a provider‐driven mechanism, but it is also a form of structural racism.^23^ We need more work to better understand the home health agencies that are serving vulnerable communities, how these home health agencies are organized, and if there are differences within the home health agency in terms of the outcomes that recipients from different backgrounds experience. It is also important to understand the home health landscape in areas with a large Hispanic/Latine population. For example, are there more for‐profit home health agencies, or are Medicaid home‐ and community‐based services more or less generous, and how do these facts impact access and outcomes for more vulnerable patient groups?

This study has several limitations and suggested future directions. First, it is not possible to establish a causal relationship without a concurrent control group that does not experience the five‐star ratings; however, our study does establish that racial/ethnic disparities worsen in the postperiod as compared with the preperiod. Second, we use the star rating from after the introduction of the stars (postperiod) to categorize home health agencies in the preperiod, which may lead to misclassification of home health agencies as high quality or non–high quality in the preperiod. However, given our sensitivity analyses and the small changes in high quality across the years, this preperiod misclassification appears to be low. Similarly, it is important that we note the lag in publicly reported data. Home health star ratings are developed using data from about 1 year prior, meaning that the reported five‐star quality rating of an agency does not necessarily reflect the actual quality of the agency at the time the patient receives care because of the necessary lag in data used for public reporting. However, we attempt to somewhat adjust for this using data reported across 2016–2018. In this way, the data from 2016 roughly represent quality in 2015, that of 2017 represent 2016, and that of 2018 approximate 2017. Future work should consider potentially recalculating quality using the raw assessment and claims data. Third, we use ZIP Code Tabulation Areas to define neighborhoods and fix the neighborhood characteristics during the study period; this geographical boundary may be considered too large. Future work would benefit from examining neighborhoods at a more granular level and accounting for the impacts of changing neighborhood characteristics on access to care. Fourth, we examine the role of neighborhood racial composition, but we do not include formal measures of residential segregation. Future work should consider using formal measures of residential segregation (e.g., dissimilarity index). Fifth, we are unable to parse out the underlying mechanisms of the observed disparities; as such, we cannot confirm if the disparate changes in access that we observe result from responses by providers, patients, or a combination of both to the five‐star ratings. Sixth, our study is limited to the initial changes that follow the introduction of the five‐star ratings, and it may be unlikely that patients and/or agencies fully understood the potential utility or impact of the stars immediately following the release. However, we do allow for some lag time of the quality of patient care star ratings, which could have allowed for more learning time, but long‐term effects may still differ and are worthy of exploration. Seventh, we do not account for the possibility that higher quality home health agencies may have capacity constraints and are unable to receive new patients in the poststar period. However, other studies have found that defining provider capacity in this home health setting is unclear, and they posit that agency capacity may not have a large impact on findings.^18^ Eighth, our analyses are limited to Medicare beneficiaries. Future work is needed to understand if racial disparities in response to public reporting exist for Medicaid home health services. In addition, these analyses do not examine the joint effects of race and income status; but from research on intersectionality, we know the intersections between race and income are important.^67^ Future work should examine the joint effects of individual race and income status and consider the role of structural intersectionality, including the experience of Black home health patients living in Black or poorer neighborhoods.^68^ Furthermore, our postperiod (2016) corresponds with the introduction of HHVBP in nine states. Although we conducted a sensitivity analysis including an indicator for HHVBP, future work should further consider the role of HHVBP in advancing equity in access to high‐quality care. Last, racial and ethnic groups are not monoliths; future work should explore different nationalities and US‐born vs. foreign‐born status within each of the groups to better understand the variability in home health agency use and the impact of policy for various groups. Additionally, future analyses should distinguish between Asians and Pacific Islanders, as they are considered distinct racial groups with diverse histories.

Despite the limitations, this is the first study to examine the impact of the home health five‐star ratings on inequities in high‐quality home health use both at an individual and neighborhood level. Although we observed a small increase in high‐quality home health agency use overall by race, income, and place, the potential impact of public reporting was disparate. Ensuring equitable access requires taking a closer look at potentially inequitable policies to ensure that these policies are not inadvertently exacerbating disparities as the home health five‐star ratings potentially do. If a reform or policy, such as the home health five‐star ratings, aims to mitigate disparities and advance equity, then policymakers and the CMS should consider adding measures of equity to the publicly reported information.^38^, ^69^, ^70^ Additional research in this area is warranted and will help inform future discussions and policies for improving access to home care services.

*Funding/Support*: This work was supported by the National Institute on Aging [1R36‐AG068199].

*Conflict of Interest Disclosures*: Authors Shekinah A. Fashaw‐Walters, Momotazur Rahman, Gilbert Gee, Maricruz Rivera‐Hernandez, CeRon Ford, and Kali S. Thomas have no potential conflicts of interest to declare. Dr. Vincent Mor is a paid consultant to NaviHealth, Inc. and chair to their Scientific Advisory Board. NaviHealth is an independent entity within Optum. The company offers postacute care management and services to more than 1.5 million beneficiaries in all regions of the country through its partnerships with health plans and health systems.

## Supporting information


**Appendix 1**. Description of Excluded Observations.
**Appendix 2**. Changes in HHA High‐Quality Status Over Time.
**Appendix 3**. Alternative Data Visualizations for Main Findings.
**Appendix 4**. Alternatively Specified High Quality Sensitivity Analyses.
**Appendix 5**. HHVBP Sensitivity Analysis. Appendix.
**Appendix 6**. Neighborhood Racial Composition Sensitivity Analysis.
**Appendix 7**. High‐Quality HHA Use by Neighborhood Poverty
