# Supplementary material for: Potentially More Out of Reach: Public Reporting Exacerbates Inequities in Home Health Access
Source: Milbank Q. 2023 Mar 24;101(2):527–59. doi: 10.1111/1468-0009.12616 (PMC10262386; doi:10.1111/1468-0009.12616)
Supplement: Supplementary file 1 — Appendix 1. Description of Excluded Observations. Appendix 2. Changes in HHA High‐Quality Status Over Time. Appendix 3. Alternative Data Visualizations for Main Findings. Appendix 4. Alternatively Specified High Quality Sensitivity Analyses. Appendix 5. HHVBP Sensitivity Analysis. Appendix. Appendix 6. Neighborhood Racial Composition Sensitivity Analysis. Appendix 7. High‐Quality HHA Use by Neighborhood Poverty [file MILQ-101-527-s001.docx]

**Supplementary Materials**

Appendix 1……………………………………………….. Description of Excluded Observations

Appendix 2…………………………………… Changes in HHA High-Quality Status Over Time

Appendix 3………………………...………… Alternative Data Visualizations for Main Findings

Appendix 4………………………….. Alternatively Specified High Quality Sensitivity Analyses

Appendix 5…………………………………………………………. HHVBP Sensitivity Analysis

Appendix 6…………………………….. Neighborhood Racial Composition Sensitivity Analysis

Appendix 7………………………………..….High-Quality HHA Use by Neighborhood Poverty

**Appendix 1- Description of Excluded Observations**

We excluded 28,386 patient assessments, representing 28,008 unique beneficiaries, because they did not include a provider number that could be matched to the CMS Care Compare to collate data on the quality of the home health agency they used. This accounted for less than 1% of our sample. There were 424 unique provider numbers included on these patient assessments that could not be matched to the Care Compare. Of the excluded patient assessments, 78% were from the pre-period, 40% were from male patients, the average age was 77.8 years, 30% were Medicare Advantage, 28% identified the patient as living alone. Thirty-eight percent of the excluded assessments were associated with low-income patients, and 63% were for white patients, 11% for Black patients, 24% for Hispanic/Latine patients, 2% for Asian American/Pacific Islander patients, and 0.4% for American Indian/Alaska Native patients. As it relates to neighborhoods, 12% of the excluded assessments were in the Northeast region of the country, 17% in the Midwest, 47% in the South, and 24% in the West. Majority of the assessments were in urban areas, 43% were in predominately white neighborhoods, 3% in predominately Black neighborhoods, 20% in predominately Hispanic/Latine neighborhoods, 11% in minority neighborhoods, and 24% in integrated neighborhoods. A quarter of the excluded assessments were in neighborhoods in the highest quintile of poverty, 15% were in the lowest quintile, 21% in the second, 18% in the middle, and 21% in the fourth poverty quintile.

**Appendix 2 – Changes in HHA High-Quality Status Over Time**

| **Characteristic** | **Percent** |
| --- | --- |
| High-Quality HHAs in 2016 | 40.4 |
| High-Quality HHAs in 2017 | 40.7 |
| High-Quality HHAs in 2018 | 43.2 |
| High-Quality Status Remained 2016-2017 | 79.1 |
| High-Quality Status Increased 2016-2017 | 10.5 |
| High-Quality Status Decreased2016-2017 | 9.6 |
| High-Quality Status Remained 2016-2018 | 70.9 |
| High-Quality Status Increased 2016-2018 | 15.5 |
| High-Quality Status Decreased2016-2018 | 11.6 |
| High-Quality Status Remained 2017-2018 | 83.4 |
| High-Quality Status Increased 2017-2018 | 8.7 |
| High-Quality Status Decreased2017-2018 | 5.9 |

**Notes.** We used the average star rating in each year to determine high-quality status. High-quality home health agencies (HHAs) have >3.5 stars.

**Appendix 3 – Alternative Data Visualizations for Main Findings**

**Figure 1B:** Percentage Point Change in High-Quality HHA, by Patient Race, Ethnicity, and Income.

**Source.** Authors’ analysis of data from the 2014-2017 Medicare Beneficiary Summary File (MBSF), the 2014-2017 Outcome and Assessment Information Set (OASIS), and the 2016-2018 Care Compare website. **Notes:** N=7,001,512 start of care assessments. Unit of Analysis: Person-Quarter Level. Adjusted analysis adjusts for sex, age, Medicare Advantage status, living along at the time of the assessment, and includes neighborhood fixed effects (FEs). High-quality home health agencies have >3.5 stars. Low-Income identifies a beneficiary as having dual enrollment in Medicare and Medicaid and/or participation in Medicare Part-D low-income cost-sharing subsidy. Medicare Advantage enrollment status as defined in the MBSF. Living Alone was measured using the OASIS variable for living situation.

**Figure 2B:** Percentage Point Change in High-Quality HHA Use for All Patients, by Neighborhood Racial Composition.

**Source.** Authors’ analysis of data from the 2014-2017 Medicare Beneficiary Summary File (MBSF), the 2014-2017 Outcome and Assessment Information Set (OASIS), and the 2016-2018 Care Compare website. Geographical data come from the 2015 American Community Survey (ACS) 5-year estimates and the 2013 National Center for Health Statistics (NCHS) Urban-Rural Classification Scheme for Counties. **Notes.** AAPI, Asian American/Pacific Islander. AIAN, American Indian/Alaska Native. Hispanic, Hispanic/Latine. LCL, Lower Confidence Level. UCL, Upper Confidence Level. High-quality home health agencies (HHAs) have >3.5 stars. Adjusted for sex, age, Medicare Advantage status, living alone, region of the country, rurality, and neighborhood poverty. Neighborhood is defined by the ZIP Code Tabulation Area. Neighborhoods that are predominately White, Black, Hispanic/Latine, AAPI, or AIAN must be made up of $\geq$65% White, Black, Hispanic/Latine, AAPI, or AIAN residents, respectively and according to the ACS. Minority neighborhoods must be made up of $\geq$65% minority residents (but not be predominately Black, Hispanic/Latine, AAPI, or AIAN), while integrated neighborhoods are ZCTAs that do not fit in the first 6 categories.

**Figure 3B:** Percentage Point Change in High-Quality HHA Use for All Patients, by Neighborhood Poverty Quintile.

**Source.** Authors’ analysis of data from the 2014-2017 Medicare Beneficiary Summary File (MBSF), the 2014-2017 Outcome and Assessment Information Set (OASIS), and the 2016-2018 Care Compare website. Geographical data come from the 2015 American Community Survey (ACS) 5-year estimates and the 2013 National Center for Health Statistics (NCHS) Urban-Rural Classification Scheme for Counties. **Notes.** AAPI, Asian American/Pacific Islander. AIAN, American Indian/Alaska Native. Hispanic, Hispanic/Latine. LCL, Lower Confidence Level. UCL, Upper Confidence Level. High-quality home health agencies (HHAs) have >3.5 stars. Adjusted for sex, age, Medicare Advantage status, living alone, region of the country, rurality, and neighborhood racial composition. Neighborhood is defined by the ZIP Code Tabulation Area. Neighborhood poverty status is operationalized using a quintile of the percent of residents who live below 200% of the Federal Poverty Line (FPL).

**Appendix 4 – Alternatively Specified High Quality Sensitivity Analyses**

We conducted sensitivity analyses with three alternatively specified high-quality outcome variables: (1) the average of the star ratings in 2016 is high quality (2) the median of the star ratings across 12 quarters is high quality, and (3) the mode of the star ratings across 12 quarters is high quality.

***The average of the star ratings in 2016 is high quality. (N=6,992,141)***

| **Adjusted Predicted Percent of High-Quality Home Health Agency Use w/ FEs** | | | | |
| --- | --- | --- | --- | --- |
| **Demographic Characteristics** | **Before Stars % (95%CI)** | **After Stars % (95%CI)** | **Difference** | **Percent Change** |
| **Race** |  |  |  |  |
| White | 40.11 | 40.64 | 0.53 | 1.32% |
|  | (40.05, 40.17) | (40.59, 40.69) | (0.46, 0.60) | (1.135 1.49) |
| Black | 38.38 | 38.66 | 0.28 | 0.73% |
|  | (38.21, 38.54) | (38.52, 38.80) | (0.09, 0.47) | (0.24, 1.22) |
| Hispanic/Latine | 41.17 | 37.12 | -4.05 | -9.84% |
|  | (40.96, 41.38) | (36.93, 37.28) | (-4.29, -3.81) | (-10.47, -9.21) |
| Asian American/Pacific Islander | 42.06 | 39.83 | -2.23 | -5.30% |
|  | (41.73, 42.39) | (39.56, 40.10) | (-2.64, -1.82) | (-6.33, -4.29) |
| American Indian/Alaska Native | 36.99 | 39.32 | 2.33 | 6.30% |
|  | (36.21, 37.76) | (38.69, 39.96) | (1.35, 3.32) | (3.73, 8.80) |
| **Income Status** |  |  |  |  |
| Higher Income | 40.15 | 40.66 | 0.51 | 1.27% |
|  | (40.09, 40.20) | (40.61, 40.71) | (.44, .59) | (1.10, 1.47) |
| Low-Income | 39.68 | 38.67 | -1.01 | -2.55% |
|  | (39.58, 39.79) | (38.58, 38.75) | (-1.14, -0.89) | (-2.88, -2.24) |
| **Overall** | 40.03 | 40.14 | 0.11 | 0.27% |
|  | (39.98, 40.08) | (40.10, 40.18) | (0.05, 0.18) | (0.13, 0.45) |

| **Percentage Point Change in High-Quality HHA use, by Neighborhood Racial Composition and Poverty** | | | | | |
| --- | --- | --- | --- | --- | --- |
| Neighborhood Type | Before Stars % | After Stars % | Difference | LCL | UCL |
| $\geq$65% White | 41.4 | 41.94 | 0.54 | 0.45 | 0.64 |
| $\geq$65% Black | 39.19 | 38.31 | -0.88 | -1.25 | -0.51 |
| $\geq$65% Hispanic/Latine | 37.95 | 31.89 | -6.06 | -6.4 | -5.72 |
| $\geq$65% AAPI | 50.45 | 44.83 | -5.62 | -8.04 | -3.2 |
| $\geq$65% AIAN | 17.68 | 20.3 | 2.62 | -1.2 | 6.45 |
| $\geq$65% Minority | 36.71 | 35.56 | -1.15 | -1.42 | -0.88 |
| Integrated | 38.43 | 38.76 | 0.33 | 0.17 | 0.48 |
| Lowest Poverty Quintile | 42.24 | 42.16 | -0.08 | -0.25 | 0.08 |
| Second Poverty Quintile | 40.16 | 40.65 | 0.49 | 0.32 | 0.65 |
| Middle Poverty Quintile | 39.71 | 39.86 | 0.15 | -0.01 | 0.32 |
| Fourth Poverty Quintile | 39.53 | 39.59 | 0.06 | -0.1 | 0.23 |
| Highest Poverty Quintile | 38.85 | 38.18 | -0.67 | -0.84 | -0.51 |

*AAPI =Asian American/Pacific Islander; AI/AN=American Indian/Alaska Native

***The MEDIAN of the star ratings across 12 quarters is high quality. (N=7,001,512)***

| **Adjusted Predicted Percent of High-Quality Home Health Agency Use w/ FEs** | | | | |
| --- | --- | --- | --- | --- |
| **Demographic Characteristics** | **Before Stars % (95%CI)** | **After Stars % (95%CI)** | **Difference** | **Percent Change** |
| **Race** |  |  |  |  |
| White | 35.52 | 36.14 | 0.62 | 1.75% |
|  | (35.46, 35.58) | (36.10, 36.19) | (.55, .70) | (1.55, 1.97) |
| Black | 33.75 | 34.63 | 0.88 | 2.61% |
|  | (33.59, 33.91) | (34.50, 34.77) | (.70, 1.07) | (2.08, 3.16) |
| Hispanic/Latine | 36.36 | 33.21 | -3.15 | -8.66% |
|  | (36.15, 36.56) | (33.04, 33.39) | (-.34, -.29) | (-.94, -.79) |
| Asian American/Pacific Islander | 36.06 | 35.07 | -0.99 | -2.75% |
|  | (35.74, 36.38) | (34.80, 35.33) | (-1.39, -.60) | (-3.89, -1.65) |
| American Indian/Alaska Native | 33.82 | 35.35 | 1.53 | 4.52% |
|  | (33.07, 34.57) | (34.74, 35.96) | (.58, 2.48) | (1.75, 7.17) |
| **Income Status** |  |  |  |  |
| Higher Income | 35.3 | 35.93 | 0.63 | 1.78% |
|  | (35.25, 35.36) | (35.88, 35.98) | (0.55, 0.70) | (1.56, 1.98) |
| Low-Income | 35.61 | 35.19 | -0.42 | -1.18% |
|  | (35.52, 35.71) | (35.1, 35.27) | (-0.55, -0.31) | (-1.55, -0.87) |
| **Overall** | 35.39 | 35.73 | 0.34 | 0.96% |
|  | (35.34, 35.43) | (35.69, 35.77) | (0.29, 0.41) | (0.82, 1.16) |

| **Percentage Point Change in High-Quality HHA use, by Neighborhood Racial Composition and Poverty** | | | | | |
| --- | --- | --- | --- | --- | --- |
| **Neighborhood Type** | **Before Stars %** | **After Stars %** | **Difference** | **LCL** | **UCL** |
| $\geq$65% White | 38.06 | 38.62 | 0.56 | 0.47 | 0.65 |
| $\geq$65% Black | 33.08 | 33.51 | 0.43 | 0.07 | 0.79 |
| $\geq$65% Hispanic/Latine | 28.7 | 23.96 | -4.74 | -5.07 | -4.41 |
| $\geq$65% AAPI | 38.66 | 36.16 | -2.5 | -4.84 | -0.17 |
| $\geq$65% AIAN | 15.43 | 17.02 | 1.59 | -2.12 | 5.28 |
| $\geq$65% Minority | 29.31 | 28.75 | -0.56 | -0.82 | -0.3 |
| Integrated | 32.61 | 33.12 | 0.51 | 0.37 | 0.66 |
| Lowest Poverty Quintile | 34.33 | 34.53 | 0.2 | 0.05 | 0.36 |
| Second Poverty Quintile | 35.12 | 35.49 | 0.37 | 0.21 | 0.53 |
| Middle Poverty Quintile | 35.72 | 35.99 | 0.27 | 0.12 | 0.44 |
| Fourth Poverty Quintile | 35.86 | 36.14 | 0.28 | 0.12 | 0.44 |
| Highest Poverty Quintile | 36.28 | 36.26 | -0.02 | -0.18 | 0.13 |

*AAPI =Asian American/Pacific Islander; AI/AN=American Indian/Alaska Native

***The MODE of the star ratings across 12 quarters is high quality. (N=7,001,512)***

| **Adjusted Predicted Percent of High-Quality Home Health Agency Use w/ FEs** | | | | |
| --- | --- | --- | --- | --- |
| **Demographic Characteristics** | **Before Stars % (95%CI)** | **After Stars % (95%CI)** | **Difference** | **Percent Change** |
| **Race** |  |  |  |  |
| White | 37.74 | 38.35 | 0.61 | 1.62% |
|  | (37.69, 37.80) | (38.30, 38.40) | (.54, .68) | (1.4, 1.79) |
| Black | 36.12 | 36.63 | 0.51 | 1.41% |
|  | (35.95, 36.27) | (36.49, 36.76) | (.33, .70) | (.92, 1.92) |
| Hispanic/Latine | 38.86 | 35.47 | -3.39 | -8.72% |
|  | (38.66, 39.07) | (35.29, 35.65) | (-3.64, -3.16) | (-9.42, -8.08) |
| Asian American/Pacific Islander | 38.91 | 37.38 | -1.53 | -3.93% |
|  | (35.29, 38.58) | (37.11, 37.65) | (-1.93, -1.13) | (-5.47, -2.93) |
| American Indian/Alaska Native | 35.81 | 37.32 | 1.51 | 4.22% |
|  | (35.05, 36.58) | (36.69, 37.94) | (.54, 2.47) | (1.54, 6.75) |
| **Income Status** |  |  |  |  |
| Higher Income | 37.52 | 38.1 | 0.58 | 1.55% |
|  | (37.46, 37.58) | (38.05, 38,15) | (0.51, 0.66) | (1.36, 1.76) |
| Low-Income | 38.04 | 37.41 | -0.63 | -1.66% |
|  | (37.94, 38.14) | (37.32, 37.49) | (-0.76, -0.51) | (-2.00, -1.34) |
| **Overall** | 37.66 | 37.92 | 0.26 | 0.69% |
|  | (37.61, 37.71) | (37.88, 37.96) | (0.2, 0.32) | (0.53, 0.85) |

| **Percentage Point Change in High-Quality HHA use, by Neighborhood Racial Composition and Poverty** | | | | | |
| --- | --- | --- | --- | --- | --- |
| **Neighborhood Type** | **Before Stars %** | **After Stars %** | **Difference** | **LCL** | **UCL** |
| $\geq$65% White | 40.29 | 40.86 | 0.57 | 0.48 | 0.66 |
| $\geq$65% Black | 35.78 | 35.74 | -0.04 | -0.4 | 0.33 |
| $\geq$65% Hispanic/Latine | 31.52 | 26.98 | -4.54 | -4.88 | -4.21 |
| $\geq$65% AAPI | 47.01 | 40.73 | -6.28 | -8.65 | -3.91 |
| $\geq$65% AIAN | 16.63 | 18.91 | 2.28 | -1.47 | 6.02 |
| $\geq$65% Minority | 31.68 | 30.53 | -1.15 | -1.41 | -0.89 |
| Integrated | 34.61 | 35.25 | 0.64 | 0.5 | 0.79 |
| Lowest Poverty Quintile | 37.01 | 36.74 | -0.27 | -0.44 | -0.11 |
| Second Poverty Quintile | 37.32 | 37.87 | 0.55 | 0.39 | 0.72 |
| Middle Poverty Quintile | 37.58 | 38.06 | 0.48 | 0.32 | 0.64 |
| Fourth Poverty Quintile | 38.02 | 38.28 | 0.26 | 0.1 | 0.42 |
| Highest Poverty Quintile | 38.56 | 38.53 | -0.03 | -0.18 | 0.14 |

*AAPI =Asian American/Pacific Islander; AI/AN=American Indian/Alaska Native

**Appendix 5 – HHVBP Sensitivity Analysis**

Given that Home Health Value-Based Purchasing (HHVBP) was introduced in 2016 (our post-period), provides a direct financial incentive to home health agencies for improving their quality, and includes measures found in the 5-star quality rating we conducted a sensitivity analysis to account for the introduction of HHVBP. We include an indicator variable for the 9 HHVBP pilot states as a covariate within the regression analysis.

| **Adjusted Predicted Percent of High-Quality Home Health Agency Use w/ FEs (including Value-Based Purchasing as a covariate)** | | | | |
| --- | --- | --- | --- | --- |
| **Demographic Characteristics** | **Before Stars % (95%CI)** | **After Stars % (95%CI)** | **Difference** | **Percent Change** |
| **Race** |  |  |  |  |
| White | 47.32 | 48.37 | 1.05 | 2.22% |
|  | (47.26, 47.37) | (48.32, 48.41) | (.977, 1.12) | (2.07, 2.36) |
| Black | 45.01 | 46.22 | 1.21 | 2.69% |
|  | (44.85, 45.18) | (46.08, 46.37) | (1.02, 1.40) | (2.27, 3.1) |
| Hispanic/Latine | 47.46 | 44.25 | -3.21 | -6.76% |
|  | (47.25, 47.68) | (44.07, 44.43) | (-3.46 -2.98) | (-7.32, -6.25) |
| Asian American/Pacific Islander | 47.24 | 46.55 | -0.69 | -1.46% |
|  | (46.91, 47.57) | (46.28, 46.82) | (-1.1 -0.28) | (-2.34, -.59) |
| American Indian/Alaska Native | 45.13 | 46.63 | 1.5 | 3.32% |
|  | (44.35, 45.90) | (45.99, 47.26) | (0.51, 2.48) | (1.15, 5.40) |
| **Income Status** |  |  |  |  |
| Higher Income | 47.18 | 48.17 | 0.99 | 2.10% |
|  | (47.12, 47.23) | (48.12, 48.22) | (.91, 1.07) | (1.93, 2.27) |
| Low-Income | 46.7 | 46.67 | -0.03 | -0.06% |
|  | (46.04, 46.81) | (46.59, 46.76) | (-.15, .09) | (-0.003, .002) |
| **Overall** | 47.06 | 47.78 | 0.72 | 1.53% |
|  | (47.01, 47.11) | (47.74, 47.82) | (0.66, 0.79) | (1.40, 1.68) |

| **Percentage Point Change in High-Quality HHA use, by Neighborhood Racial Composition and Poverty (including a Value-based Purchasing Covariate)** | | | | | |
| --- | --- | --- | --- | --- | --- |
| **Neighborhood Type** | **Before Stars %** | **After Stars %** | **Difference** | **LCL** | **UCL** |
| $\geq$65% White | 49.35 | 50.79 | 1.44 | 1.34 | 1.53 |
| $\geq$65% Black | 46.31 | 47.02 | 0.71 | 0.34 | 1.09 |
| $\geq$65% Hispanic/Latine | 39.43 | 34.73 | -4.7 | -5.04 | -4.35 |
| $\geq$65% AAPI | 52.04 | 50.03 | -2.01 | -4.44 | 0.43 |
| $\geq$65% AIAN | 25.19 | 28.9 | 3.71 | -0.15 | 7.56 |
| $\geq$65% Minority | 40.93 | 41.3 | 0.37 | 0.1 | 0.64 |
| Integrated | 43.97 | 45.51 | 1.54 | 1.39 | 1.7 |
| Lowest Poverty Quintile | 47.41 | 47.91 | 0.5 | 0.34 | 0.67 |
| Second Poverty Quintile | 46.86 | 47.78 | 0.92 | 0.75 | 1.086 |
| Middle Poverty Quintile | 47.2 | 47.92 | 0.72 | 0.55 | 0.88 |
| Fourth Poverty Quintile | 47.23 | 47.59 | 0.36 | 0.193 | 0.52 |
| Highest Poverty Quintile | 47.2 | 47.25 | 0.05 | -0.11 | 0.22 |

*AAPI =Asian American/Pacific Islander; AI/AN=American Indian/Alaska Native

Appendix 6 – Neighborhood Racial Composition Sensitivity Analysis

Given the relatively large size of our neighborhoods (identified by ZIP Code Tabulation Areas) we conduct a sensitivity analysis lowering the neighborhood racial composition measurement from >65% to >50%.

| Percentage Point Change in High-Quality HHA use, by Neighborhood Racial Composition and Poverty | | | | | |
| --- | --- | --- | --- | --- | --- |
| Neighborhood Type | Before Stars % | After Stars % | Difference | LCL | UCL |
| >50% White | 48.71 | 49.62 | 0.91 | 0.82 | 0.99 |
| >50% Black | 45.75 | 46.2 | 0.45 | 0.16 | 0.75 |
| >50% Hispanic | 40.4 | 36.98 | -3.42 | -3.69 | -3.15 |
| >50% AAPI | 49.71 | 49.35 | -0.36 | -1.46 | 0.74 |
| >50% AIAN | 27.6 | 28.37 | 0.77 | -2.43 | 3.96 |
| Minority | 42.55 | 43.48 | 0.93 | 0.69 | 1.16 |
| Integrated | 44.95 | 44.92 | -0.03 | -0.45 | 0.39 |

Appendix 7 – High-Quality HHA Use by Neighborhood Poverty

**
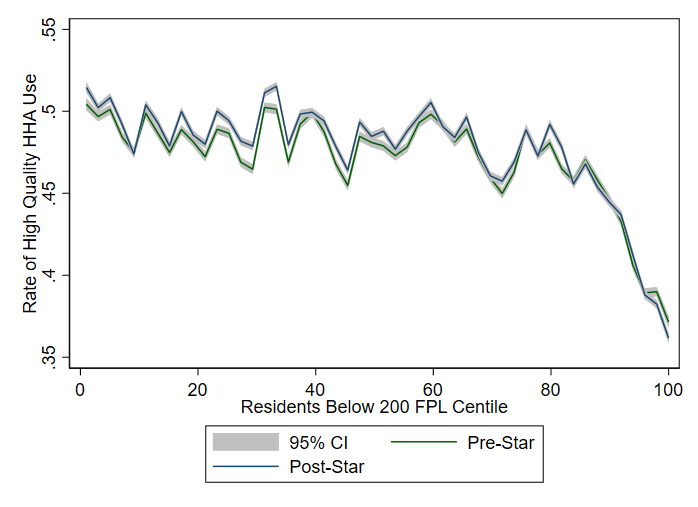
**
